# Effects of Shen-Fu Injection on the Expression of T-Cell-Specific Transcription Factors T-bet/Gata-3 in Porcine Postresuscitation Lung Injury

**DOI:** 10.1155/2013/464650

**Published:** 2013-03-13

**Authors:** Wei Gu, ChunSheng Li, WenPeng Yin, XiaoMin Hou, Da Zhang

**Affiliations:** Department of Emergency Medicine, Beijing Chaoyang Hospital, Capital Medical University, Beijing 100020, China

## Abstract

Shen-Fu injection (SFI) derived from the ancient traditional Chinese medicine. In this study, the effects of SFI on the expression of T-bet/GATA-3 and its potential mechanisms causing the shift of T cells from Th2 to Th1 on postresuscitation lung injury were examined in a porcine model of cardiac arrest. 30 pigs were randomly divided into SHAM (*n* = 6) and three return of spontaneous circulation (ROSC) groups (*n* = 8 per group); 24 pigs were subjected to 8 min of electrically induced cardiac arrest and 2 min of basic life support, which received central venous injection of Shen-Fu (SFI), epinephrine (EP) or saline (SA). After successful ROSC, 18 surviving pigs were sacrificed at 24 h after ROSC (*n* = 6 per group). The levels of serum and lung tissue interleukin (IL)-4 and interferon (IFN)-**γ** were measured by ELISA, and the protein and mRNA levels of GATA-3 and T-bet in the lung tissue were determined by western blotting and quantitative real-time polymerase chain reaction, respectively. Compared with the EP and SA groups, SFI treatment reduced the levels of IL-4 (*P* < 0.05), increased levels of IFN-**γ** (*P* < 0.05), and induced T-bet mRNA upregulation and GATA-3 mRNA downregulation (*P* < 0.05). SFI attenuated lung injury and regulated lung immune disorders. Therefore, SFI could protect postresuscitation lung injury by modulating a Th1/Th2 imbalance.

## 1. Introduction

Cardiac arrest (CA) represents the most severe shock state, during which the delivery of oxygen and metabolic substrates is abruptly halted. Increased oxygen debt leads to endothelial activation and results in the systemic release of pro- and anti-inflammatory mediators [1], such as tumour necrosis factor-*α* (TNF-*α*), which promotes immune suppression and systemic inflammation [2], thereby contributing to postresuscitation lung injury. However, the mechanisms responsible for postresuscitation lung injury are not well understood. Several other mechanisms have been not only hypothesized but shown to be involved, including oxygen-derived-free radicals, ischemia/reperfusion, and apoptosis [3–5]. 

The balance of T helper type 1 (Th1) and T helper type 2 (Th2) cells is important in regulating immune function and inflammatory response. Th1 lymphocytes produce IFN-*γ* and interleukin (IL)-2, favouring cell-mediated immunity; Th2 lymphocytes secrete IL-4, IL-5, IL-10, and IL-13, favouring humoral immunity [6]. As Th1 and Th2 cells are differentiated from common T precursor cells, their differentiation requires the activity of specific transcription factors, including T-bet and GATA-3 [6, 7]. Thus, changes in the expression levels of GATA-3 and T-bet contribute to an imbalance in Th1 and Th2 cells. Protective immunity is dependent on the proper balance of Th1 and Th2 cells [8]. Alterations in the correct balance of Th1/Th2 cells are associated with a series of immune and inflammatory diseases, such as burn or trauma, acute myocardial infarction, heart failure, and severe sepsis [9–13]. 

Shen-Fu injection (SFI) derived from the ancient traditional chinese medicine and mainly composed of Ginsengand Fuzi extracts (Ya'an Sanjiu Pharmaceutical Co., Ltd., China). Its quality is controlled strictly according to the standard of China Ministry of Public Health, and fingerprint technology was adopted in the process of production to ensure its quality. Some studies have proved that SFI had protective effect on acute lung injury induced by endotoxin and shock [14, 15]. Our previous work also showed that SFI can attenuate postresuscitation lung injury by modulating apoptosis [5]. However, the other mechanisms responsible for the lung protective effects of SFI are unknown. Therefore, in this study, we established a swine model of CA, in which we examined the effects of SFI on the expression of T-bet and GATA-3, and found that SFI after ROSC could reduce postresuscitation lung injury by modulating lung Th1/Th2 imbalance.

## 2. Materials and Methods

The study was conducted with the approval of The Capital Medical University Institutional Animal Care Committee, and all animals received treatments in compliance with the National Research Council's 1996 Guide for the Care and Use of Laboratory Animals.

### 2.1. Animal Preparation

Thirty inbred male Wuzhishan miniature pigs (12–14 months of age, 30 ± 2 kg) were used in this study. Pigs were randomly divided into 4 groups, resuscitation groups: Shen-Fu injection (SFI), epinephrine (EP), saline (SA) (*n* = 8, per group), and sham operation (SHAM) (*n* = 6). Animals were fasted overnight but were allowed free access to water. After premedication with 0.5 mg/kg intramuscular midazolam, anesthesia was induced by ear vein injection of propofol (1.0 mg/kg) and maintained in a surgical plane of anesthesia with intravenous infusion of pentobarbital (8 mg/kg/h) [15]. Heart rate and electrocardiogram measurements were monitored using a four-channel physical recorder (BL-420F Data Acquisition & Analysis System; Chengdu TME Technology Co, Ltd, China). A cuffed 6.5 mm endotracheal tube was advanced into the trachea. Animals were mechanically ventilated with a volume-controlled ventilator (Servo 900C; Siemens, Germany) with a tidal volume of 15 mL/kg and FiO_2_ of 0.35 using a tidal volume of 15 mL/kg and a respiratory frequency of 12/min. An angiographic catheter was inserted from the femoral artery into the aortic arch to measure aortic pressure. The electrocardiogram and all hemodynamic parameters were monitored with a patient monitoring system (M1165; Hewlett-Packard, Palo Alto, CA). 

### 2.2. Experimental Protocol

After surgery, the animals were allowed to equilibrate for 30 min to achieve a stable resting level. The temporary pacemaker conductor was inserted into the right ventricle through the right sheathing canal and connected to an electrical stimulator (GY-600A; Kaifeng Huanan Equipment Co., Ltd., China) programmed in the S1S2 mode (300/200 ms), 40 V, 8 : 1 proportion, and 10 ms step length to provide a continuous electrical stimulus until ventricular fibrillation (VF) [16]. VF was defined as an electrocardiogram showing waveforms corresponding to VF and a rapid decline in mean aortic pressure toward zero [17]. Ventilation was stopped while inducing VF, and ventilation was withheld for the entire 8-minute duration of VF arrest. After 8 min of VF, manual CPR was carried out at a frequency of 100 compressions/min with mechanical ventilation at an FiO_2_ of 100% and a compression-to-ventilation ratio of 30 : 2. The quality of chest compressions was controlled by a HeartStart MRx Monitor/Defibrillator with Q-CPR (Philips Medical Systems, Best, Holland) [16]. After 2 minutes of CPR, pigs were randomized divided into 3 groups, then receive, respectively, central venous injection of Shen-Fu injection (1.0 mL/kg) (SFI), epinephrine (0.02 mg/Kg) (EP) and saline (SA). If the spontaneous circulation was not restored, defibrillation was attempted once using a diphase 150 J. If spontaneous circulation was still not achieved, CPR was continued for a further 2 minutes and defibrillation was attempted once more. The same procedure without CA initiation was achieved in the SHAM group, including induction of anaesthesia, electrode positioning, mechanical respiration, 8 mins ventilation withheld, and monitoring of physiological parameters. 

ROSC was defined as 10 consecutive min of maintenance of systolic blood pressure at 50 mm Hg. If spontaneous circulation was not restored within 30 min, we regarded the animal as dead [17]. The survival animals were euthanized with 10 mL of 10 mol/L potassium chloride intravenously at 24 hrs after resuscitation. The left external jugular vein was cut open, the catheter was put into the right common carotid artery and normal saline was infused through the catheter smoothly into the artery until the blood became clear, then the chest was opened, and the right middle lung lobe was separated and obtained. 

### 2.3. Measurements

Arterial blood samples were taken at baseline, 0, 30 min, 2, 6 hours after ROSC; furthermore, venous blood samples were taken at baseline, 30 min, 6, 12, and 24 hours after ROSC. Arterial blood gas was examined (GEM Premier 3000 blood gas analyzer, Instrumentation Laboratory, Lexington, MAs). Some lung specimens were preserved in 4% paraformaldehyde to observe pathologic changes for light microscope and transmission electron microscopy, whereas others were stored at −80°C for enzyme-linked immunosorbent assay (ELISA), quantitative real-time PCR, and western blotting.

### 2.4. The Measurement of Lung W/D

The left upper lobe of the lungs was obtained, the blood and water of the lung surface were removed by using absorbent paper to get the “wet” weight (W), and then the wet lung was put into stoving chest at 70°C for 72 hrs to get the “dry” weight (D). The ratio of the wet lung to the dry lung was calculated to assess tissue edema. 

### 2.5. BALF Total Cell and PMN Counts

The pigs were anesthetized again at 24 h after ROSC and provided with a plastic cannula inserted into the trachea. BALF was performed with three aliquots of 5 mL PBS (pH7.2) instilled up to a total volume of 15 mL and withdrawn three times each. The recovery rate of BALF was 95%. BALF samples were centrifuged (400 ×g, 4°C) for 5 min. The sediment cells were resuspended in 100 *μ*L PBS. The total BALF cells were counted double blindly using a hemocytometer followed by the differential counting of leukocytes (Giemsa staining; two counts per slide, 300 cells per count). 

### 2.6. Th1 and Th2 Related Cytokines Analyses

IL-4 (6R019) and IFN-*γ* (6R082) in the serum and lung tissue were measured by ELISA according to the manufacturers'instructions (Sunbio Biotech Co. Ltd., China). 100 mg lung tissue and 2 mL vein blood were, respectively, centrifuged at 35,000 ×g for 10 min at 4°C, supernatants and centrifuged blood were collected and immediately frozen at −80°C for ELISA, microtitration plates were coated overnight with 50 uL of a 10 ug/mL solution of Biotin anti- IL-4 and INF-*γ* in carbonate buffer pH 9.6. After blocking and washing, the sample was incubated for 30 min at room temperature. Plates were washed and added with 50 *μ*L HRP, then, incubated for 30 min again at room temperature, and subsequently, the plates were washed again. Each well was added with 50 *μ*L color liquid, gently mixed for 30 seconds, and incubated for 15 minutes at room temperature. The reaction was stopped after 15 min by the addition of 50 *μ*L stop solution. The absorbance at 450 nm was measured after 30 min using an ELISA plate reader. Cytokine concentrations were obtained from a standard curve. Duplicate readings were obtained for all samples and the means were calculated. 

### 2.7. Western Blot Analysis of GATA-3 and T-bet

A 150-mg frozen lung sample was homogenized in 2 mL of ice-cold buffer. The lung tissues were homogenized and then centrifuged at 12,000 r/min for 10 min at 4°C. A total of 100 *μ*g of protein was loaded onto 10% SDS-PAGE gel in each sample. Western blotting was performed with the membranes blocked for 2 h with 5% nonfat milk and then incubated with the primary antibodies (diluted overnight at 4°C): GATA-3, 1 : 500 (ab113519; Abcam Biotechnology, UK); T-bet, 1 : 200 (sc-21003; Santa Cruz Biotechnology, USA); and GAPDH, 1 : 250 (Santa Cruz Biotechnology, USA). Blots were blocked and incubated at 4°C overnight with the specific primary antibody. The immunoreactive bands were visualized on film and scanned. The data were analysed by Image Pro Plus (Version 4.1, Media Cybernetics). The quantitative data from Western blot bands were expressed as the target protein OD/GAPDH OD ratio.

### 2.8. Quantitative Real-Time PCR Assay for GATA-3 and T-bet

Total RNA was extracted from 50 to 100 mg of lung tissue according to the protocol described for the BioEasy SYBR Green I Real-Time PCR Kit Manual (Bo Ri Technology Co., Ltd., China). Preincubation was performed at 95°C for 2 min, followed by amplification in 45 cycles at 95°C for 20 s, 59°C (GATA-3 and T-bet), 72°C for 30 s, and finally, during slow heating up, 72°C for 10 min. After the amplification, melting curve analysis with a temperature gradient from 65°C to 95°C was recorded every 0.5°C (hold for 5 s). The primer sequences of the expected PCR products were as follows: for GATA-3 (240 bp), Forward primer: 5′-TGCGGGCTCTACCACAAAAT-3′ and reverse primer: 5′-TAACCCGAGTAAAATGTGC-3′; for T-bet (118 bp), forward primer: 5′-ACAAACCCGATATGGCTGAGA-3′ and reverse primer: 5′-CCTGCTTGCTTCTCCTGTTC-3′; and for GAPDH, Forward primer: 5′-CCATCACTGCCACTCAGAAGACT-3′, and Reverse primer: 5′-GTCAGATCCACAACGGATACATTG-3′. Relative quantification is generally calculated with the 2^−ΔΔCT^ formula by the comparative Ct method, the copy number of the target gene 2^−ΔΔCT^ = 2^−(ΔCT  target  gene  −  ΔCT  GAPDH  gene)^ [17].

### 2.9. Statistical Analysis

The experimental data were analyzed by SPSS 17.0 (SPSS Inc., Chicago, IL, USA). The results are expressed as mean ± SD, and Student's *t* test was used for comparisons between two groups. Differences at different time points within groups were compared with repeated-measures ANOVA. A two-tail value of *P* < 0.05 was considered significant.

## 3. Results

18 of 24 animals were successfully resuscitated. By comparison, the number of electric shock, defibrillation energy, and time to ROSC were significantly lower in EP and SFI group than that in SA group (*P* < 0.05, Table 1), but there was no difference in EP and SFI groups (*P* > 0.05, Table 1).

### 3.1. Blood Gas Analysis

PH and PaO_2_ in the post-ROSC group decreased more significantly than those in the sham-operated group within post-ROSC 6 h (Table 2), Furthermore, PH and PaO_2_ in the SFI group increased more significantly than those in the EP and SA groups (Table 2). 

### 3.2. Ultrastructural Changes in Lung Tissue

**Figure 1:**
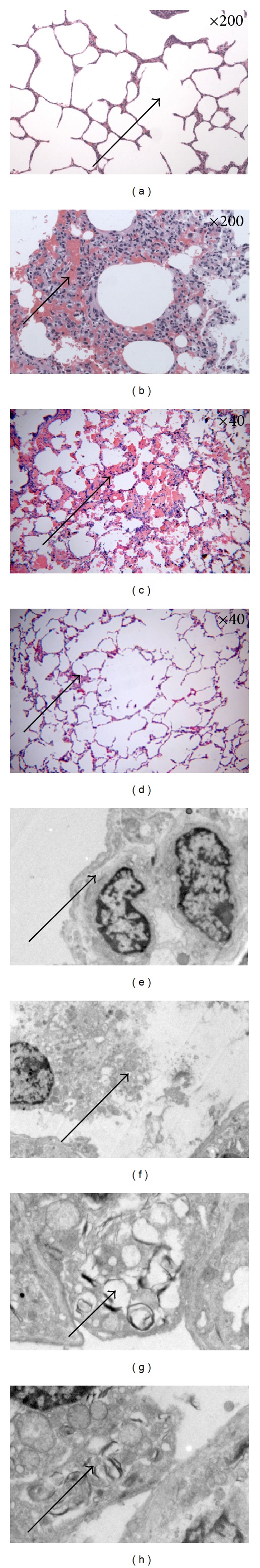
Lung histopathology photographs. (a)–(d) Light microscope images (HE, ×200); (e)–(h) electron microscope images; (a) and (e) photographs from the sham-operated group; (b), (c), (f), and (g) photographs from the EP group; (d) and (h) photographs from Shen-Fu injection group. (b) and (c) Pulmonary arterioles contain erythrocytes, oedema fluid, and inflammatory cells (arrows). (d) Congestion, inflammatory cell infiltration and edema of the lung tissue were considerably alleviated (arrows). (e) normal type II epithelial cells structure was observed in the SHAM group (arrows). (f) Type II epithelial cells are heavily injured, exhibiting necrosis and disintegration (arrows). (g) The structure of the lamellar bodies in type II epithelial cell presents vacuolar change (arrows) inside the cell. (h) The lamellar body of type II epithelial cells shows minor damage in SFI group (arrows).

By light microscopy, lung tissue structure appeared normal in the SHAM group (arrows) (Figure 1(a)), whereas it was severely damaged in the EP and SA groups. The alveolar cavity was narrow, the alveolar wall became thick, and the capillary of alveolar wall dilated. There was obvious blood and edema fluid exudation in alveolar cavity. There were also exfoliated epithelial cells and neutrophil infiltration in alveolar cavity. (Figures 1(b) and 1(c)). Congestion, inflammatory cell infiltration, and edema of the lung tissue were considerably alleviated with SFI treatment (Figure 1(d)). Using electron microscopy, normal type 2 epithelial cells structure was observed in the SHAM group (arrows) (Figure 1(e)). Epithelial cells turned into necrosis and disintegration, and the lung tissue exhibited progressive, severe deterioration in the EP and SA groups 24 h after cardiac resuscitation (arrows) (Figure 1(f)). The lamellar body (characteristic structures of type II epithelial cells, which produce pulmonary surfactant to reduce alveolar surface tension) of type 2 epithelial cells cracked (arrows) (Figure 1(g)). Animals treated with SFI exhibited minimal type 2 epithelial cells structure damage in the lung tissue (Figure 1(h)).

### 3.3. The Change of Lung W/D Ratio

The lung W/D ratio was markedly increased in the SA, EP, and SFI groups (6.27 ± 0.31, 5.93 ± 0.29, and 3.89 ± 0.27) compared with that in the SHAM group (2.41 ± 0.21) (*P* < 0.01), while the lung W/D ratio was significantly decreased in the SFI group compared with the SA and EP groups at 24 h after ROSC (*P* < 0.05) (Table 3).

### 3.4. BALF Total Cell and PMN Counts

As illustrated in Table 4, the post-ROSC group showed a remarkably higher BALF total cell and PMN counts when compared with sham-operated group (*P* < 0.01). Furthermore, the SFI group showed a remarkably lower BALF total cell and PMN counts when compared with SA and EP groups (*P* < 0.01).

### 3.5. ELISA Analysis of IL-4 and IFN-*γ* Levels

**Figure 2:**
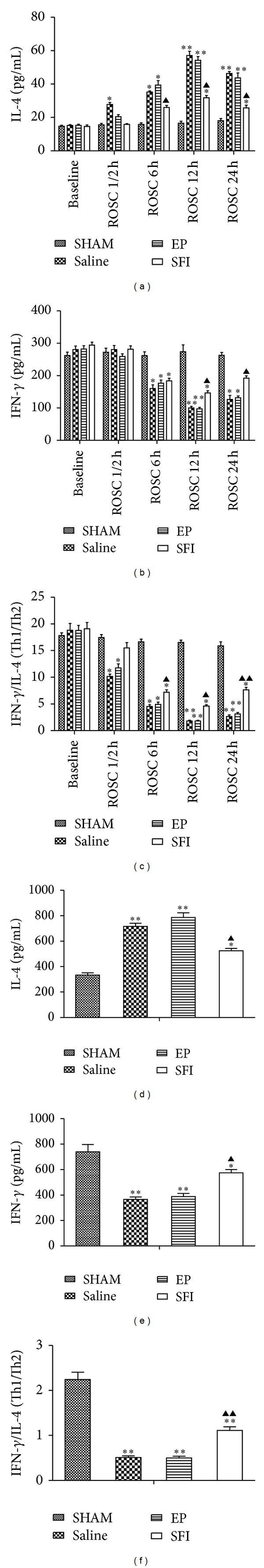
ELISA analysis of (a)–(c) IL-4, IFN-*γ*, and IFN-*γ*/IL-4 levels in the serum; (d)–(f) IL-4, IFN-*γ*, and IFN-*γ*/IL-4 levels in the lung tissue: significant difference (*P < 0.05; **P < 0.01 versus SHAM) between the SHAM group, SA, EP, and SFI group (*n* = 6), ^▲^ *P* < 0.05, ^▲▲^ *P* < 0.01 versus EP and SA.

The results showed that the levels of serum IL-4 were markedly increased at 1/2-24 h after ROSC, while IFN-*γ* and IFN-*γ*/IL-4 (Th1/Th2) were significantly decreased in the SA, EP, and SFI groups compared with those in the SHAM group (*P* < 0.05 or *P* < 0.01) at 6–24 after ROSC. Particularly, the levels of serum IL-4 at 12 h were higher than those at all time points after ROSC (*P* < 0.01); however, the levels of serum IFN-*γ* were at 12 h lower than that at all time points after ROSC (*P* < 0.01) (Figure 2). Furthermore, the levels of lung tissue IL-4 were markedly increased in the SA, EP and SFI groups compared with those in the SHAM group (*P* < 0.05 or *P* < 0.01) at 24 h after ROSC, while IFN-*γ* and IFN-*γ*/IL-4 (Th1/Th2) were significantly decreased, and the levels of lung IL-4 were markedly decreased in the SFI group compared with those in the SA and EP groups (*P* < 0.05 or *P* < 0.01) at 24 h after ROSC, whereas IFN-*γ* and IFN-*γ*/IL-4 (Th1/Th2) were markedly increased (Figure 2). 

### 3.6. Protein Expressions of GATA-3 and T-bet

**Figure 3:**
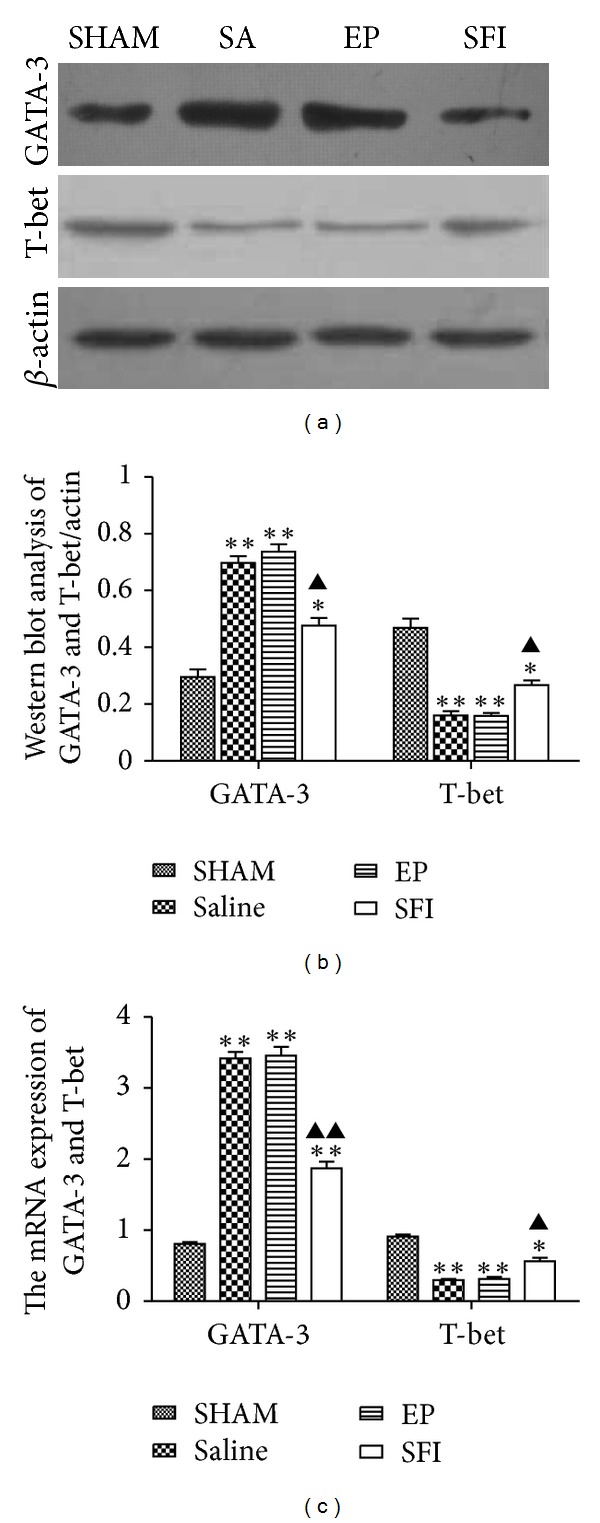
ELISA analysis of IL-4, TNF-*α*, and IFN-*γ* levels in the lung tissue: (a) western blots of GATA-3 and T-bet protein expressions in the lung tissue at 24 h after ROSC. (b) Western blots of quantification of GATA-3 and T-bet protein levels. (c) mRNA expressions of GATA-3 and T-bet in the lung tissue at 24 h after ROSC. The values represent mean ± SE (*n* = 6) *P < 0.05 and **P < 0.01 versus SHAM; ^▲^ *P* < 0.05, ^▲▲^ *P* < 0.01 versus EP and SA. (one-way repeated-measures ANOVA).

As shown in Figure 3, western blot analysis showed that GATA-3 expression progressively increased in the lung tissue (*P* < 0.05), while the expression of T-bet progressively decreased in the SA, EP and SFI groups compared with the SHAM group at 24 h after ROSC. Furthermore, GATA-3 expression was significantly decreased, while T-bet was significantly increased in the SFI group compared with the EP and SA groups at 24 h after ROSC (*P* < 0.05). 

### 3.7. The mRNA Levels of GATA-3 and T-bet

The mRNA levels of the GATA-3 gene were significantly increased (*P* < 0.01), while the mRNA levels of the T-bet gene were significantly decreased in the SA, EP, and SFI groups compared with those in the SHAM group (*P* < 0.01). Furthermore, the mRNA levels of GATA-3 in the SFI group were reduced compared with those in the EP and SA groups (*P* < 0.05) at 24 h after ROSC, whereas the mRNA levels of T-bet were increased (*P* < 0.05) in the SFI group (Figure 3).

## 4. Discussion

During CA, a compensatory increase in systemic oxygen extraction occurs, which leads to significantly decreased central or mixed venous oxygen saturation [18]. Inadequate tissue oxygen delivery can persist even after ROSC; accumulated oxygen debt leads to activation of immunologic pathways and systemic inflammation [1], which increases the risk of multiple organ failure [19–21]. The specific structural and functional characteristics of lung tissue make it very vulnerable even after circulation has been successfully restored. In the present study, pigs that underwent CA all presented with severe post-resuscitation lung injury, manifested by severe histopathological damage, low oxygenation index, high BALF total cell and PMN counts and increased lung W/D ratio. Furthermore, an shift from the Th1 to Th2 response induced by the change in expression levels of the transcription factors GATA-3 and T-bet in the lung tissue occurs in postresuscitation lung injury. 

Our results show that SFI group had a better-histopathological change, decreased lung W/D ratio, high oxygenation index, and low BALF total cell and PMN counts compared to the EP and SA groups, suggesting that SFI can reduce postresuscitation lung injury. “SFI decoction” has been commonly used in China for nearly 800 years. SFI is a typical form of SFI decoction for intravenous medication, whose main components include ginsenoside (0.8 mg/mL) and aconitine (0.1 mg/mL) [5]. Some studies have also shown that the effects of SFI are based on aconitine properties, supplemented by ginsenoside, which can scavenge free radicals, improve energy metabolism [22], inhibit inflammatory mediators [23], suppress cell apoptosis [5], and alleviate mitochondrial damage [24]. A previous study undertaken in our laboratory showed that SFI attenuated postresuscitation lung injury by inhibiting lung cell apoptosis, and by improving energy metabolism and antioxidant capacity [5].

In actual study, the expression levels of IFN-*γ* and IL-4 depend on the activity of Th1 and Th2 cells. IFN-*γ*, which is generated by activated T and NK cells, can promote Th0 cells to differentiate Th1 cells and inhibit the proliferation of Th2 cells [25]. IL-4 can stimulate and activate the proliferation of B and T cells, promote the differentiation of Th0 cells into Th2 cells and inhibit the differentiation of Th0 cells into Th1 cells. In our study, post-ROSC pigs exhibited a change in their Th1/Th2 cytokine profile in the serum and lung tissue, which was characterized by an increase in IL-4 and a decrease in IFN-*γ*. During the early postresuscitation period, CA contributes to hemodynamic disorders that cause the systemic release of massive oxygen free radical, lactic acid, and metabolites of arachidonic acid, which could reach the lung tissues by blood circulation and cause ischemia-reperfusion injury. These inflammatory factors are also interactive and make the inflammatory response lose control, which contribute to a lung imbalance of Th1/Th2. This imbalance promotes lung immune suppression and improves development of lung injury. Furthermore, this lung imbalance of Th1/Th2 could increase the risk of multiple organ failure and infection [19], This condition had many features in common with sepsis [20].

 In some previous study, the immunomodulatory effects of SFI could (1) reduce the expression of TNF-*α* to block the vicious circle of inflammatory response, (2) improve systemic microcirculation, prolong the hypoxia tolerance duration, increase blood supply and oxygenation, and promote the release and application of oxygen, (3) inhibit xanthine oxidase directly, protect the SOD activity, and resist lipid peroxidation, (4) ginsenoside could block the cell calcium channel to prevent calcium overload, it could also promote the synthesis of pulmonary surfactant and improve oxygenation, (5) promote the liver detoxifying function to reduce the endotoxin caused lung injury [14, 15, 26]. Furthermore, a previous study demonstrated that ginsenoside could stimulate immune function, improve both specific and nonspecific immunity, and influence both humoral immunity and cell immunity. They showed that total ginseng soap could promote the biosynthesis of proteins [27]. fuzi could also improve immune function by enhancing the adrenocortical system [26]. Our results also show that SFI group could improve lung immune function by modulating the Th1/Th2 imbalance. Thus, SFI could reduce postresuscitation lung injury and improve lung immune function by regulating lung imbalance of Th1/Th2.

GATA-3, a member of the GATA family of zinc-finger transcription factors, has been identified as a key regulator of Th2 cell development [28]. In contrast, T-bet, a member of the T-box family of transcription factors, is a key controller of Th1 cell differentiation [28]. GATA-3 specifies Th2 lineage commitment by inducing lineage-restricted target genes, such as IL-4, whereas T-bet specifies Th1 lineage commitment by inducing genes for IFN-*γ* [28, 29]. Correspondingly, a increase in GATA-3 could lead to IL-4 production, whereas T-bet leads to suppression of IFN-*γ* production. In the present study, both the protein expression and mRNA levels of GATA-3 were markedly reduced, while T-bet was significantly increased in the lung tissue of pigs in the SFI group compared with that in the EP groups. SFI can therefore promote the secretion of the Th1 cytokine IFN-*γ* by upregulating the expression of T-bet, and inhibit the Th2 cytokine IL-4 by downregulating GATA-3 in the lung tissue. Thus, SFI can modulate the lung imbalance of T-bet and GATA-3 expression levels and correct and restore the balance of lung Thl and Th2 immune cells. Furthermore, our results show that SFI has certain advantages compared with EP and SA, not only improving lung histopathological change and reducing lung injury, but also modulating lung Th1/Th2 imbalances by regulating changes in the expression of the transcription factors GATA-3 and T-bet in the lung tissue.

## 5. Conclusion

Our results demonstrate that SFI could improve lung immune function and protect from postresuscitation lung injury by modulating Th1/Th2 cells imbalances in the lung tissue compared with EP and SA.

## Figures and Tables

**Table 1:** Resuscitation outcome.

Group	Number of shock	Energy of shock (J)	Time to ROSC (min)
SA	5.5 ± 2.5	795 ± 375.68	10.0 ± 3.79
EP	2.75 ± 1.66*	312.7 ± 134.28**	6 ± 2.17**
SFI	2.61 ± 1.03^∗▴^	332.5 ± 168.39^∗∗▴^	5 ± 1.69^∗∗▴^

*P < 0.05, **P < 0.01 versus SA (one-way repeated-measures ANOVA); ^▴^ *P* > 0.05, versus EP (student's *t* test).

**Table 2:** Changes of arterial blood gas (PH, PaO_2_, PaCO_2_, and PaO_2_/FiO_2_) at baseline, 0, 30 min, 2 h, and 6 h after ROSC in SHAM, SA, EP and SFI groups (mean ± SD).

Group	baseline	ROSC 0 min	ROSC 30 min	ROSC 2 h	ROSC 6 h
PH					
SHAM	7.42 ± 0.05	7.44 ± 0.03	7.46 ± 0.11	7.38 ± 0.09	7.41 ± 0.14
SA	7.42 ± 0.12	7.05 ± 0.17**	7.11 ± 0.19**	7.19 ± 0.23*	7.31 ± 0.11
EP	7.39 ± 0.12	7.01 ± 0.29**	7.03 ± 0.18**	7.08 ± 0.27*	7.28 ± 0.19
SFI	7.41 ± 0.21	7.02 ± 0.13**	7.23 ± 0.24^∗∗▴^	7.28 ± 0.27^∗▴^	7.38 ± 0.27
PaO_2_					
SHAM	100.9 ± 6.4	93.3 ± 5.7	97.5 ± 4.1	98.9 ± 7.1	104.2 ± 6.1
SA	98.4 ± 4.9	41.4 ± 6.1**	59.8 ± 3.1*	64.9 ± 2.8*	75.7 ± 2.1
EP	100.4 ± 3.8	38.4 ± 5.3**	53.5 ± 2.8*	58.1 ± 3.6*	69.7 ± 3.7
SFI	98.4 ± 4.9	37.8 ± 5.2**	60.1 ± 4.9*	72.6 ± 2.4^∗▴^	83.1 ± 2.6^▴^
PaCO_2_					
SHAM	36.1 ± 2.6	35.2 ± 1.9	34.3 ± 3.7	37.1 ± 2.1	38.1 ± 1.9
SA	35.9 ± 1.4	49.7 ± 4.4*	44.7 ± 3.8	42.2 ± 2.5	37.5 ± 3.6
EP	36.4 ± 2.8	53.7 ± 3.6*	47.7 ± 4.1	46.1 ± 3.3	39.8 ± 2.9
SFI	37.4 ± 1.7	55.2 ± 3.6*	43.6 ± 3.1	44.1 ± 4.2	38.8 ± 3.5
PaO_2_/FiO_2_					
SHAM	293.2 ± 35.6	278.1 ± 16.2	282.8 ± 19.5	292.8 ± 11.7	299.7 ± 9.9
SA	299.5 ± 22.1	114.8 ± 18.7**	178.5 ± 19.3*	181.4 ± 16.8*	207.6 ± 8.5
EP	302.1 ± 17.2	107.8 ± 15.9**	155.5 ± 13.4*	176.1 ± 19.8*	200.1 ± 7.9
SFI	297.5 ± 18.3	109.8 ± 17.6**	188.5 ± 9.8*	209.6 ± 14.1^∗▴^	247.6 ± 9.1^▴^

The values represent mean ± SE (*n* = 6) *P < 0.05 and **P < 0.01 versus SHAM; ^▴^ *P* < 0.05 versus EP and SA.

**Table 3:** The chang of lung W/D ratio at 24 h after ROSC in the SHAM, SA, EP, and SFI groups (mean ± SD).

	SHAM	SA	EP	SFI
Lung W/D ratio	2.41 ± 0.21	6.27 ± 0.31**	5.93 ± 0.29**	3.89 ± 0.27^∗▴^

The values represent mean ± SE (*n* = 6) *P < 0.05 and **P < 0.01 versus SHAM; ^▴^ *P* < 0.05 versus EP and SA.

**Table 4:** The change of BALF total cell (×10^7^) and PMN counts (×10^7^) at 24 h after ROSC in the SHAM, SA, EP and SFI groups (mean ± SD).

	SHAM	SA	EP	SFI
BALF total cell	0.56 ± 0.11	6.11 ± 0.65*	7.02 ± 0.39*	4.33 ± 0.19^∗▴^
BALF PMN counts	0.49 ± 0.09	4.11 ± 0.21*	5.66 ± 0.29*	2.91 ± 0.31^∗▴^

The values represent mean ± SE (*n* = 6) *P < 0.05 versus SHAM; ^▴^ *P* < 0.05 versus EP and SA.
